# Low cost tips for tip-enhanced Raman spectroscopy fabricated by two-step electrochemical etching of 125 µm diameter gold wires

**DOI:** 10.3762/bjnano.9.254

**Published:** 2018-10-22

**Authors:** Antonino Foti, Francesco Barreca, Enza Fazio, Cristiano D’Andrea, Paolo Matteini, Onofrio Maria Maragò, Pietro Giuseppe Gucciardi

**Affiliations:** 1 CNR-IPCF, Istituto per i Processi Chimico-Fisici, Viale F. Stagno D’Alcontres 37, 98168 Messina, Italy; 2 Dipartimento di Scienze Matematiche e Informatiche, Scienze Fisiche e Scienze della Terra, Università degli Studi di Messina, Viale F. Stagno d’Alcontres 31, 98166 Messina, Italy; 3 IFAC-CNR, Institute of Applied Physics “Nello Carrara”, National Research Council, Via Madonna del Piano 10, 50019 Sesto Fiorentino, Italy

**Keywords:** amyloid, enhanced spectroscopy, gold tips, plasmonics, TERS

## Abstract

Tip-enhanced Raman spectroscopy (TERS) has become a well-applied technique for nanospectroscopy, allowing for single molecule sensitivity with sub-nanometer spatial resolution. The demand for efficient, reproducible and cost-effective probes for TERS is increasing. Here we report on a new electrochemical etching protocol to fabricate TERS tips starting from 125 µm diameter gold wires in a reproducible way. The process is reliable (50% of the tips have radius of curvature <35 nm, 66% <80 nm), fast (less than 2 min) and 2.5 times cheaper than the etching of standard 250 µm diameter wires. The TERS performance of the tips is tested on dyes, pigments and biomolecules and enhancement factors higher than 10^5^ are observed. TERS mapping with a spatial resolution of 5 nm is demonstrated.

## Introduction

Tip-enhanced Raman spectroscopy (TERS) combines the chemical and structural information of Raman spectroscopy with the large signal gain provided by plasmonic resonances in metal tips and the high spatial resolution mapping offered by scanning probe microscopy [1–5]. In TERS, sharp metallic (or metallized) tips act as optical nanoantennas [6–7]. The tips efficiently enhance and confine the electromagnetic field at the nanoscale [8–9] or even at sub-nanometer levels [10]. TERS has a sensitivity that can reach the single molecule level [11–12]. TERS setups based on atomic force microscopy (AFM) [1,13], scanning tunneling microscopy (STM) [14] and shear-force microscopy (ShFM) [15] allow for chemical imaging of nanostructured materials, surfaces and (bio)molecular layers with a spatial resolution of 4–10 nm in ambient conditions [15–16], and can even reach atomic-level sensitivity in ultrahigh vacuum (UHV) [17–19]. Excellent reviews on the applications of TERS can be found in [20–25]. TERS features unique advantages as compared with scanning electron microscopy (SEM), scanning near-field Raman microscopy (Raman-SNOM) [26] and far-field nanoscopy [27–28]: (i) it is a label-free technique, i.e., it does not require sample pretreatment, (ii) it can be operated in ambient conditions, liquid environments, as well as in UHV and at low temperatures, (iii) it combines the surface morphology information with the chemical information, (iv) optical excitation powers are virtually unlimited, and (v) it can attain atomic-level resolution. The presence of commercial setups on the market has further increased the application of TERS outside of the traditional chemistry and physics laboratories, suggesting TERS could be used as a future routine characterization tool like AFM, UV–vis, Raman or FTIR spectroscopies.

The tip is the key element in TERS. Its field enhancement and confinement capabilities determine the signal amplification, the spatial resolution and the reproducibility of the results. The material, morphology, aspect ratio and size of the tip apex are expected to determine the optical properties of the tip [29–30]. TERS tips are nowadays produced by the chemical/electrochemical etching of metal wires [31–35], metal coatings of AFM tips [36–38], electroless deposition, [39] galvanic displacement [40] or by advanced nanostructuration techniques such as electron beam induced deposition (EBID) and focused ion beam (FIB) milling [41–43] (see [30,44] for reviews). Fabrication methods capable of guaranteeing high reproducibility, cost-effectiveness and scalability to industrial production are, however, still not available at present. Metal vapor deposition on AFM tips is intrinsically scalable and tips of any kind of material can be used, but the reproducibility is low and the field enhancement is not excellent. Nanofabrication methods guarantee optimal control of the dimensions and reproducibility, but they are serial techniques, i.e., slow, and fabrication costs are high. In addition, all the above-cited methods require very expensive lab equipment and skilled operators. Electrochemical etching, although suffering from surface roughness issues (mainly for silver), reproducibility issues and lack of tip dimension control, is a technique that is easy to implement, accessible to every laboratory, and requires low-cost equipment and minimum training of the personnel. When applied to gold, electrochemical etching yields tips with good surface quality and a small radius of curvature in minutes, at reasonable costs, that can be safely stored for months [31,45–46]. Smooth tips with a radius of curvature smaller than 50 nm are reliably obtained with >80% success rate by etching 250 µm gold wires at low voltages (≈2.4 VDC, in order to avoid bubbling in the etching solution) by controlling the current [31], or monitoring the etching time [46].

Decreasing the diameter of the gold wire is a way to reduce the costs. The price of a tip can be calculated as the sum of the price of the gold wire plus that of the chemicals needed for the etching. We do not consider the cost of the labor here, since it can be highly variable depending on whether the operator is a diploma student or a technician/researcher. At the current market prices, a stock (5 m) of gold wire with 250 µm diameter costs 820–910 €, compared to 350–410 € for the same length of 125 µm diameter wire (Advent/Goodfellow). The average cost per tip (typically 1.0–1.5 cm long) ranges between 1.7–2.4 €/tip using 250 µm wires, and 0.7–1.1 €/tip using 125 µm wires. The cost of the chemicals is also different. In our experience, with 30 mL of HCl/ethanol solution, we can prepare up to five tips by etching 250 µm wires, whereas 10 mL are enough for 125 µm wires, corresponding to 0.45 €/tip and 0.15 €/tip, respectively. The final average cost per tip is thus 2.50 €/tip for 250 µm wires, against 1.05 €/tip for 125 µm wires.

In this article we report on a new protocol to produce TERS tips by electrochemical etching of 125 µm gold wires. The protocol tailors a two-step procedure [46] in which the first step is carried out at high voltage to quickly shrink the wire diameter and the second one is carried out at low voltage, in smooth, bubble-free conditions. Smooth TERS-effective tips are obtained in 80% of the cases. Tips with a radius of curvature of 35 nm are obtained with a 50% success rate, with etching times of approximately 2 min. The tips can be easily manipulated and safely mounted, by gluing or clamping them into STM- or ShF-based TERS setups. The good performance of the tips is highlighted by TERS spectra of dyes, pigments and biomolecules. The enhancement factor in the range of 10^4^–10^5^ was found. Finally, a spatial resolution of ≈5 nm is shown on TERS maps of rhodamine 6G (R6G) sub-monolayers absorbed onto gold monocrystals.

## Experimental

**Figure 1:**
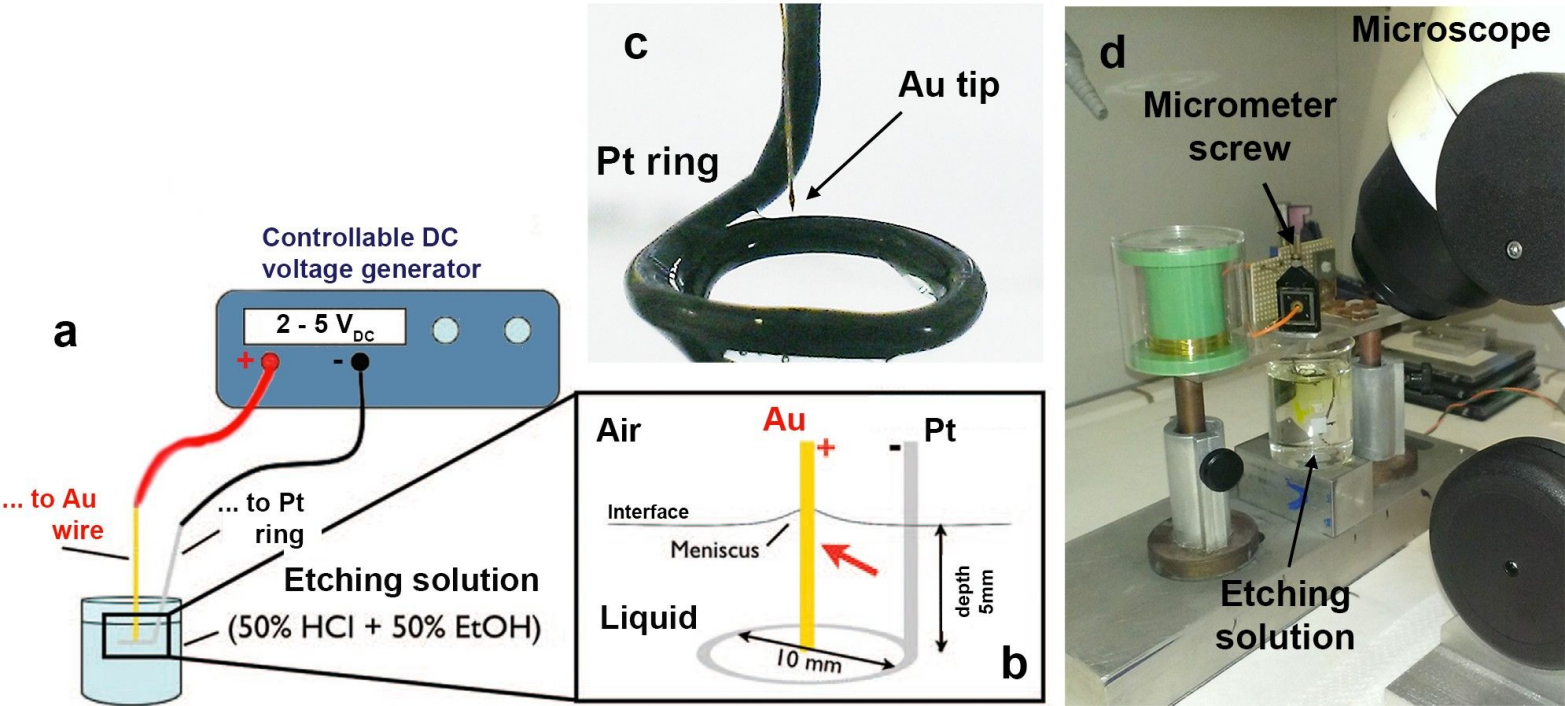
(a) Experimental setup. (b) Details of the tip immersion zone, highlighting the geometry and position of the electrodes. (c) Photograph of the electrodes. (d) Photograph of the overall setup.

Gold wires (125 μm diameter, Advent AU517311, high purity 99.99%, temper hard) are etched in a 10 mL solution 1:1 v/v of fuming hydrochloric acid (>37 wt %) and absolute ethanol (>99.5 wt %). All the reagents used are of analytical grade. The experimental setup is shown in Figure 1.

**Figure 2:**
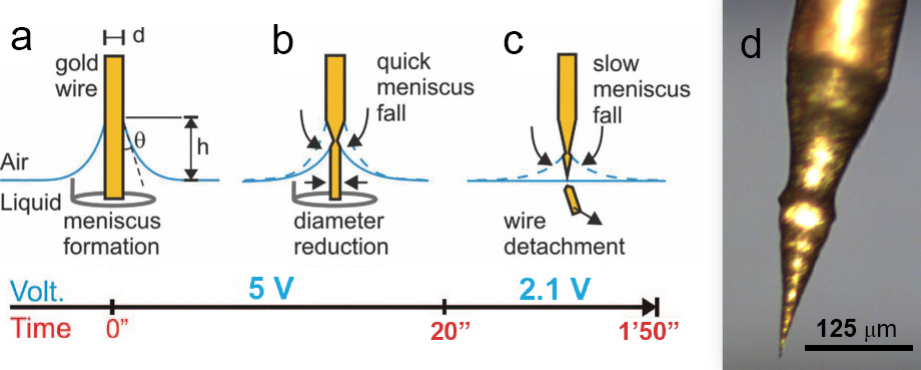
Temporal evolution of the two step electrochemical etching process. (a) A meniscus is formed at the air–liquid interface when the wire is immersed in the solution. (b) The meniscus height decreases rapidly during the high-voltage etching (5 V_DC_) due to the wire thinning. This phase is accompanied by intense bubbling. (c) The process slows down when the voltage is lowered to 2.1 V_DC_ and the etching proceeds without bubbles. The process self-terminates with the detachment of the immersed portion of the wire that leaves a sharp gold tip at the end. (d) Optical image of a typical gold tip, shaped from a gold wire of 125 µm diameter.

The setup includes (Figure 1a) an adjustable DC voltage generator connected to (Figure 1b) the gold wire to be etched (the anode) and to a platinum wire (500 µm diameter, Advent PT5408, temper hard) shaped to form a ring of 10 mm diameter, acting as the cathode. A micrometric translator is used to manipulate the gold wire during the immersion and extraction from the solution. The gold wire is placed at the center of the ring-shaped cathode (Figure 1c) and oriented orthogonal to the liquid surface. Both electrodes are dipped 5 mm below the air–liquid interface (Figure 1b). The etching process is inspected with a stereo microscope (Figure 1d) mounted with a CCD camera (Thorlabs). The etching is carried out in two steps as depicted in Figure 1a–c and shown in Supporting Information File 1. When we immerse the gold wire in the ethanol–HCl solution we observe the formation of a meniscus at the metal–air–liquid interface, due to capillary forces (Figure 2a) [47].

A pre-etching step of the gold wire is performed at a voltage *V*_1_ = 5 V_DC_ for a time *t*_1_ = 15–20 s (Figure 2b) and permits quick reduction of the wire diameter and, consequently, almost halves the overall tip production time. During this step the wire diameter at the meniscus is thinned at a rate δ*d*/δ*t* ≈ 3.5–4 μm/s and intense bubbling is observed. As a rule of thumb, the pre-etching should be limited to 20 s in order to prevent a reduction of the diameter below 40–50 μm, which would make it too fragile and subject to bending or early detachment. Bubbling, in fact, intensely shakes the wire portion protruding into the solution (red arrow in Figure 1b). Any bending or early detachment of this wire part would yield a crooked or blunt tip. The second etching step is carried out by lowering the DC voltage in the 2.1–2.3 V range (Figure 2c). No bubbling occurs under these conditions. The tip slowly forms at the air–liquid interface, with an etching rate δ*d*/δ*t* ≈ 0.5–1.5 μm/s depending on the exact voltage applied (the higher the voltage, the higher the etching rate). The process self-terminates when the portion of the wire immersed in the solution detaches (Figure 2c), which typically occurs after *t*_tot_ = 60–150 s. A wire with a double taper and a sharp tip at the end is thus obtained (Figure 2d). The voltage is turned off immediately after the precipitation of the immersed wire portion in order to avoid over-etching, which would blunt the tip. Stop-voltage circuits have been developed [48–49] to automatize this task. The tips are finally washed by shaking in the etching solution and, subsequently, by pouring a few drops of HCl and rinsing in ethanol and water. This eliminates residual impurities from the surface.

Scanning electron microscopy (SEM) inspection of the produced tips is carried out to characterize the tip apex using a Zeiss Merlin field emission electron microscope, equipped with a Gemini II column.

The analysis of the tips’ TERS performance is carried out in gap-mode [14], using a commercial setup that couples a micro-Raman spectrometer (XploRA Plus, Horiba) with an AFM/STM (Smart SPM-1000, AIST-NT). The setup, shown in Supporting Information File 2, works in a side-illumination configuration with a 638 nm laser beam, *p*-polarized, focused onto the tip axis through a 100× long working distance objective (Mitutoyo, WD 6.0 mm, NA 0.7), oriented at 60° with respect to the vertical axis. The backscattered TERS signal is collected by the same objective. The signal is dispersed by a grating featuring 1200 gr/mm and sent to a Peltier cooled CCD camera (Syncerity, Horiba Jobin Yvon). The laser spot is positioned on the tip apex with the aid of a piezoelectric *x*–*y*–*z* table that scans the objective position. The *x*–*y* scan plane is orthogonal to the optical axis (*z*) of the objective.

## Results and Discussion

### Gold etching and tip formation mechanism

The gold electrochemical corrosion is driven by a well-known redox process in acidic environment [31], whose main reactions are:

[1]
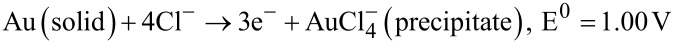


[2]
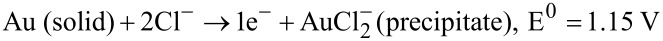


[3]
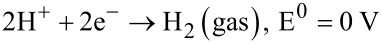


Here the superficial gold atoms are oxidized, transforming into either Au(I) or Au(III). The chlorine ions combine with Au(I) or Au(III) (reactions 1 and 2), yielding a yellow precipitate. At the same time, H^+^ ions are reduced at the platinum wire surface, leading to H_2_ gas formation (reaction 3). The H_2_, together with O_2_ and Cl_2_ present in the solution, can cause intense bubbling when the reaction is fast enough, as for example at 5 V_DC_ (first step of the process). At lower voltages (2.1–2.2 V_DC_), as in the second step of the process, the reaction proceeds much more slowly. The ethanol acts effectively as a quencher, hindering any production of bubbles. The tip formation is ruled by the meniscus lowering consequent to the wire thinning under electrochemical attack [46,50]. The etching process is not homogeneous along the wire profile immersed in solution. The etching at the meniscus is ≈1.5 times faster than in the bulk. As can be seen in Supporting Information File 1, at the beginning of the low voltage step the wire diameter at the meniscus is thinner than the portion immersed in the liquid. This difference is likely due to the anisotropic distribution of the reaction products (AuCl^4−^ and AuCl^2−^) along the wire surface that hinders the renewal of fresh etchant. The reaction products generated at the meniscus fall due to gravity, covering the surface of the wire still immersed in the bulk solution, affecting the concentration distribution of the chlorine ions along the wire. A higher local concentration of Cl^−^ ions at the meniscus with respect to the bulk can justify the observed anisotropic etching rate.

### Morphological analysis

**Figure 3:**
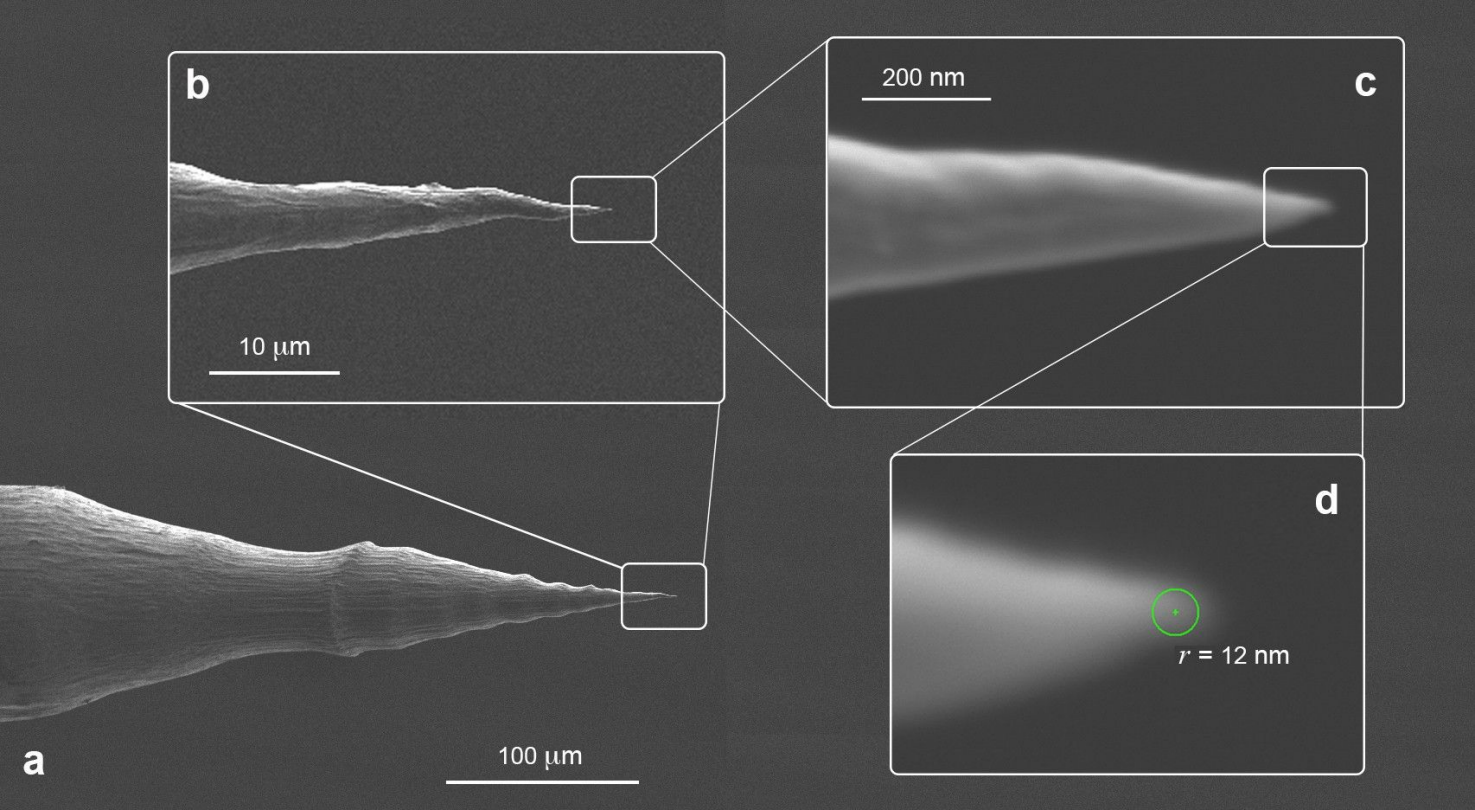
(a) SEM images of a pilum-shaped tip with details shown in (b), (c) and (d) on the apical part, showing a radius of curvature *r*_tip_ ≈ 12 nm and an apical angle of ≈24° (*t*_1_ = 20 s, *V*_1_ = 5 V_DC_, *t*_2_ = 90 s, *V*_2_ = 2.1 V_DC_).

The tip morphology was characterized using SEM. Figure 3a (details shown in (b, c)) show a typical pilum*-*shaped tip obtained after the two-step etching process, ending with a radius of curvature of *r*_tip_ ≈ 12 nm (d).

**Figure 4:**
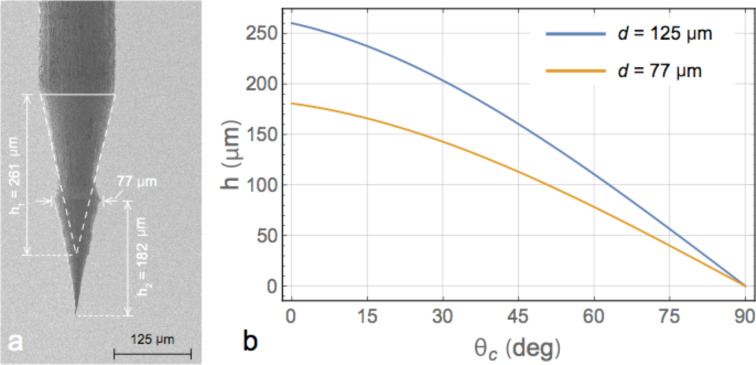
(a) SEM image of a tip highlighting the dimensions of the tapered zones. The dashed lines are a contour of the upper taper that would have been obtained in a single step etching. (b) Plot of the expected meniscus height as a function of the contact angle assuming a wire diameter of 125 µm (blue line, upper taper) and of 77 µm (orange line, lower taper).

A double taper, ≈350 μm long (Figure 4a) tip is observed, resulting from the two etching steps. The overall tip length is a factor of two shorter with respect to that observed on 250 µm diameter Au wires. The lower taper (≈180 μm) features an apical angle of ≈24° and ends with a sharp tip. The lower taper is also characterized by a wavy profile, which smooths towards the apex region (Figure 3c). This is a consequence of the burst-like behavior observed during the etching process. The tip length and its aspect ratio are determined by the meniscus height *h* (Equation 4), where *h* depends on the wire diameter, *d*, and contact angle, θ_c_ according to [46]

[4]
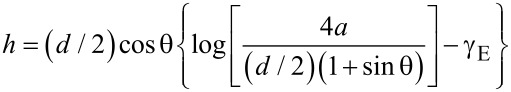


where γ_E_ ≈ 0.577 is Euler’s constant, *g* = 981 cm/s^2^ is the gravitational constant and 
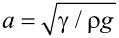
 is the capillary constant, where γ ≈ 30 dyn/cm and ρ = 0.98 g/cm^3^ are the surface tension and the density of the HCl/ethanol solution, respectively. In Figure 4b we plot the predicted values of the meniscus extension *h* as a function of the contact angle for a starting diameter of 125 µm (blue line) and 77 µm (orange line), corresponding to the initial wire size and to the wire diameter at the beginning of the second etching step. The meniscus height *h*_2_ predicted for the second etching step assuming very small contact angles (θ_c_ ≈ 0, Figure 4b, orange line) matches very well with the taper length (*h*_2_ = 182 µm, Figure 4a). The overall tip length found in the experiments (≈360 µm) is, however, longer than meniscus height *h*_1_ = 260 µm predicted for a wire diameter of 125 µm, even for θ_c_ ≈ 0 (Figure 4b, blue line). The value of *h*_1_, however, matches remarkably well the length of the tip expected if we had completed the etching at high voltage (261 µm, Figure 4a, dotted line). This observation suggests that the length discrepancy can be attributed to the bubbling phenomenon. As visible in Supporting Information File 1, bubbles originating from the wire immersed in the solution reach the surface and explode. The effect is particularly intense in proximity of the liquid–air–metal interface. As a consequence, the motion of the bubbles results in a force that raises the meniscus contact point with the metal wire with respect to the equilibrium conditions described by Equation 4. We believe that this phenomenon is at the origin of the ≈100 µm meniscus upshift and overall tip length increase.

**Figure 5:**
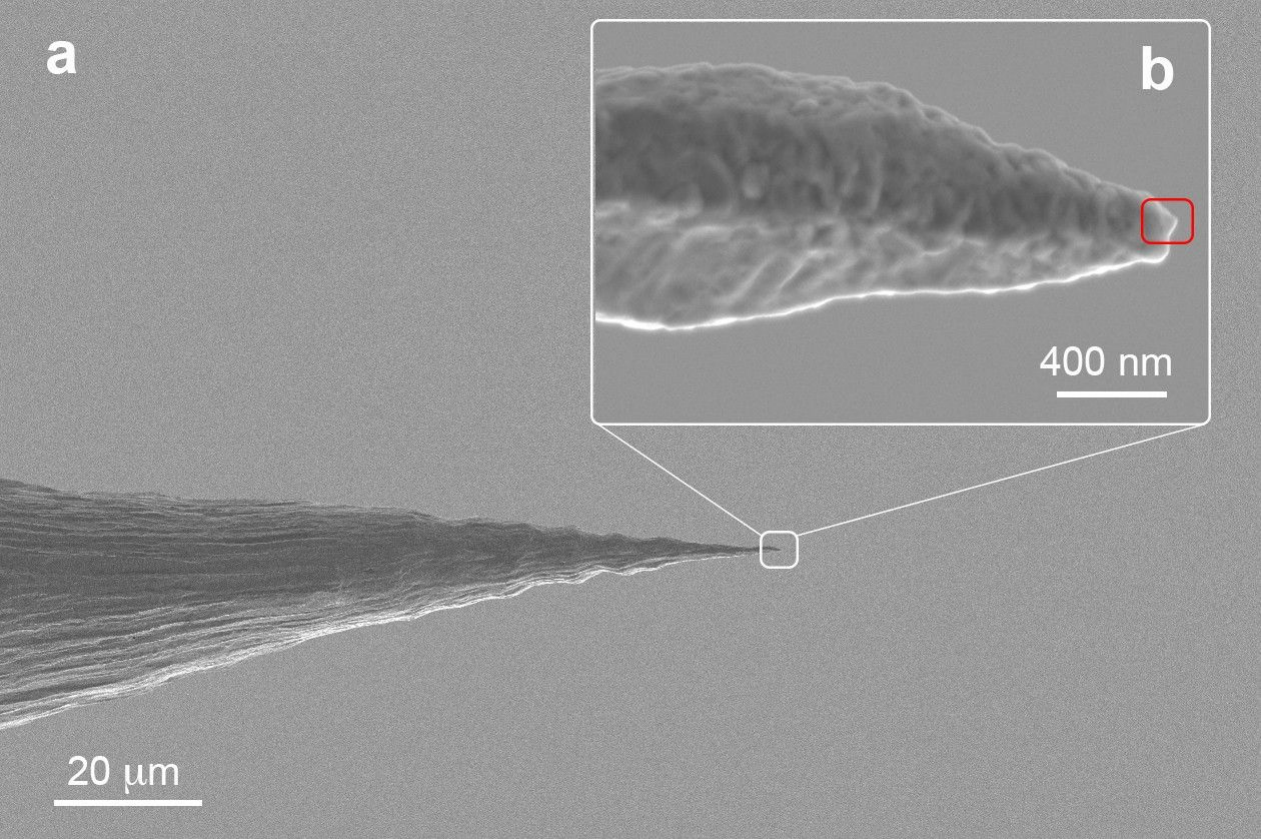
(a) SEM image of an etched tip (*t*_1_ = 20 s, *V*_1_ = 5 V_DC_, *t*_2_ = 75 s, *V*_2_ = 2.1 V_DC_) and details on its apex (b), showing the roughness of the gold surface. Sharp protrusions (*r* ≈ 15 nm) can occur at the apex of such blunt tips (red square).

The intrinsic morphology of the metal wires plays a key role in determining the final tip characteristics [30]. We find that when randomly etching different sections of the gold wire supplied by the producer, we end up with very rough tips (Figure 5), even in the presence of “fresh” solutions. Rough surfaces have been explained [46] with the presence of dislocations and grains in the 100–500 nm scale (consequence of the wire production process), in which the etching occurs through the detachment of large pieces of gold, instead of in a smooth atom-by-atom fashion. Sharp protrusions are occasionally observed at the apex of rough tips. At present it is not possible to predict and control such phenomenon. A statistical analysis on ten tips shows that 80% of the tips are TERS-active, while 50% of the tips have a radius of curvature smaller than 35 nm.

### Light emission from the tip apex

Tightly coupled plasmonic metals, such as nanorods dimers [51], nanocubes on surfaces [52], or TERS tips in contact with surfaces [53–54], emit light over a broad continuum, even if excited at energies below the *sp*/*d* interband transition. Enhanced inelastic electron tunneling through the gap seems to be the origin of photon emission [52,55], leading to electronic Raman scattering (ERS) of the laser photons [53] which is at the origin of the background observed in TERS and SERS [54–56]. The spectral features of such a light continuum bring information on the plasmonic modes of the nanoantenna system. For processes concerning single tips, i.e., withdrawn from the substrates, the origin of the light emission has not yet been unambiguously ascertained. Calculations on nanocube monomers [53] predict a 100-fold smaller light emission with respect to the nanocube-on-surface configuration, and the origin of the signal is attributed to photoluminescence rather than to ERS. Sanders et al. [57], working on Au-coated spherical AFM tips, have shown a remarkable correlation between the dark-field scattering peak, attributed to plasmon excitations, and the position of the maximum integrated SERS background of the tip, confirming that the background is enhanced by the localized plasmon resonance in the apical region. On sharp Au tips, the same authors report an almost flat scattering (from the visible to the NIR), associated with a less intense background in the 600–700 nm region. In any case, it is a matter of evidence that a stronger background is typically associated to a more intense SERS/TERS emission [54,57], suggesting the presence of a more effective substrate/tip near-field coupling.

**Figure 6:**
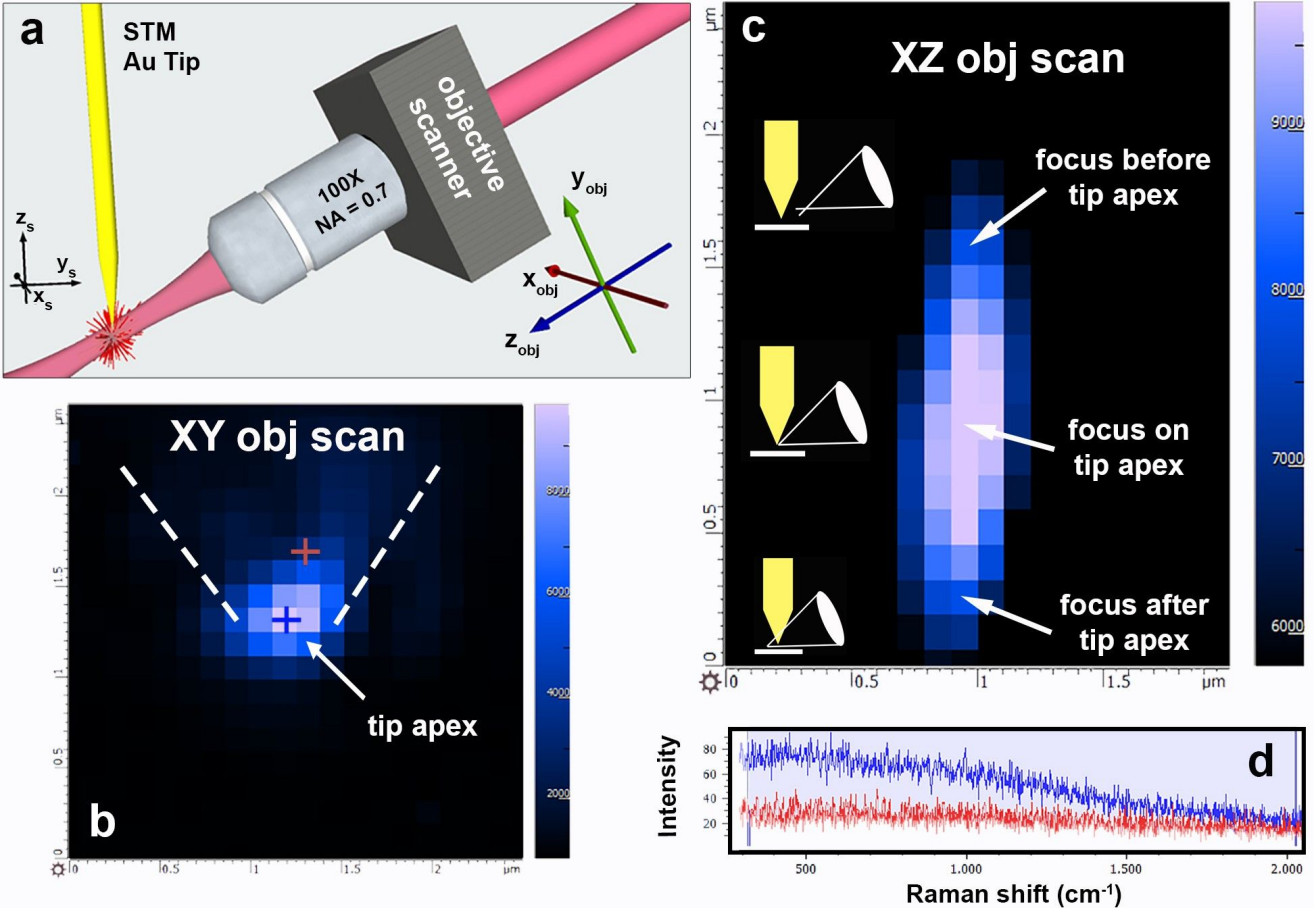
(a) Sketch of the scanning configuration for the mapping of the light emitted by the tip. The tip is in sample reference frame (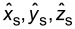). The laser spot is scanned through a 3D piezo actuator that moves the objective in the (
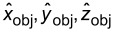
) reference frame, allowing us to carry out scans in both the (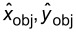) and in the (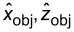) planes. (b) *x*–*y* fluorescence map of a gold tip (2.5 × 2.5 µm^2^, step size 125 nm). The dotted lines highlight the shape of the tip. (c) *x–z* fluorescence map of the gold tip (1.9 × 2.4 µm^2^, step size 100 nm). The pictograms illustrate the different focusing conditions. Each pixel represents the integrated intensity from 300 to 2000 cm^−1^. (d) Spectra acquired at the apex of the tip (blue line) and on the shaft (red line) correspond to the positions highlighted with the blue and red crosses in (b). Laser power *P* = 1 mW, integration time per pixel *t* = 0.5 s.

For our purposes, mapping the light emission from the tip is important for two reasons. Firstly, we have empirically verified that optimal tips for TERS are those that show some degree of light emission from their apex_._ Secondly, the process allows us to precisely focus the laser spot on the tip apex, maximizing the overall TERS signal. Measurements were carried out by scanning the laser spot with a piezoelectric stage attached to the objective (Figure 6a).

Two maps are acquired: one to localize the tip apex position (Figure 6b) in which we scan the laser spot in the (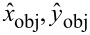) plane that, given the large incidence angle, is almost coincident with the tip plane (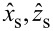); the second one is to optimize the laser focus on the tip apex (Figure 6c), and is carried out by scanning the laser beam in the (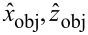) plane, i.e., moving the beam orthogonally to the tip axis while changing the focusing conditions. In each map we report the intensity of the background signal (Figure 6d) integrated in the 300–2000 cm^−1^ range. Spectra acquired inside (blue cross) and outside (red cross) the apical region show the presence of a continuum background (Figure 6d, blue line) at the apex, compared to a flat signal on the shaft (red line). Typical laser powers are 1.0–2.5 mW and integration times are 0.5–1.0 s per pixel.

### TERS spectra of dyes, pigments and biomolecules

**Figure 7:**
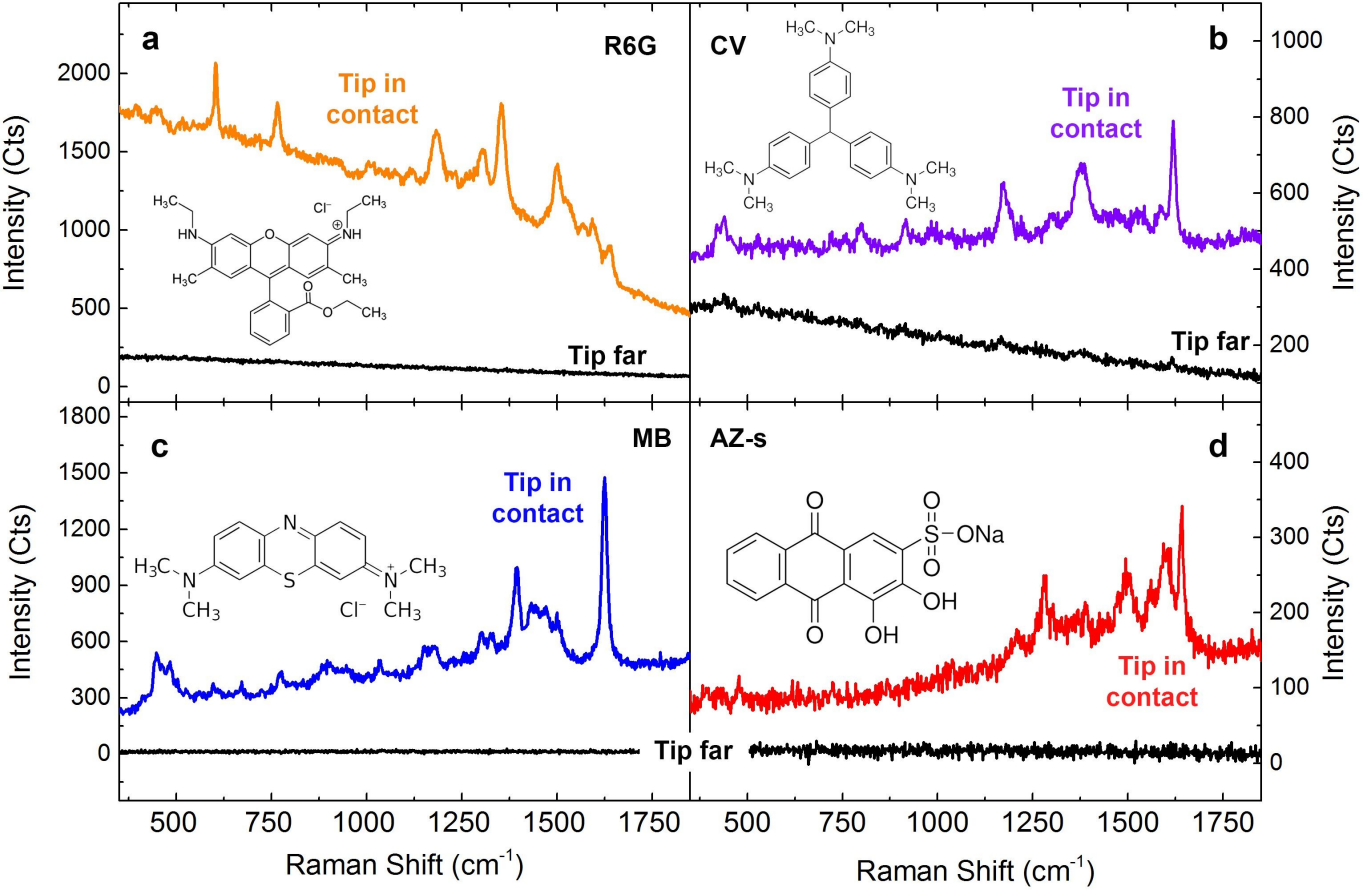
TERS spectra (colored lines) of different molecules acquired with the tip in contact with the surface: (a) R6G (*P* = 1 mW, *t* = 5 s), (b) CV (*P* = 1 mW, *t* = 3 s), (c) MB (*P* = 0.1 mW, *t* = 1 s), (d) AZ-s (*P* = 1 mW, *t* = 5 s). Black lines represent signal intensity acquired in the same conditions when the tip is far from the sample.

The tips have been applied to evaluate the spectra of analyze standard dye molecules such as rhodamine 6G (R6G), crystal violet (CV), methylene blue (MB), pigments of cultural heritage interest (alizarin-s, AZ-s) [58] and highly toxic protein oligomers [59]. Tests are carried out in gap-mode, absorbing the probe molecules on gold films with side-illumination at an excitation wavelength of 638 nm. Molecular solutions at different concentrations are prepared in deionized water. Target molecules are absorbed on Au(111) flat films that have undergone standard flame annealing in order to obtain crystalline terraces of about 100 nm in size. The gold film substrates are immersed for 2 h and 30 min and subsequently rinsed in deionized water in order to remove the molecules excess. Finally, they are dried under a nitrogen flux. Figure 7 shows the TERS spectra acquired on R6G at 10^−4^ M (a), CV at 10^−5^ M (b), MB at 10^−5^ M (c) and AZ-s at 10^−3^ M (d).

**Figure 8:**
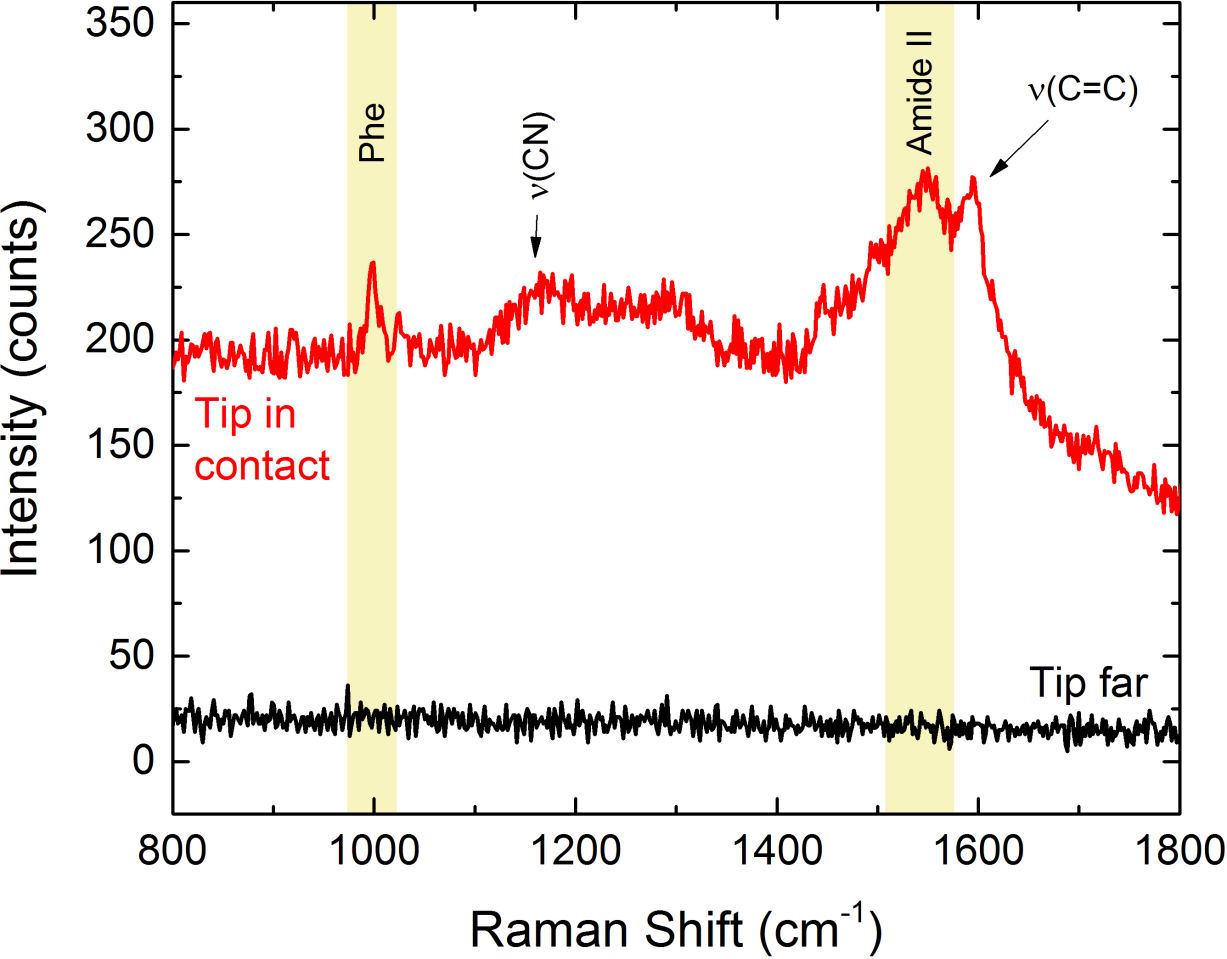
TERS signal of oligomers when the STM feedback loop is on (red line) and when it is off (black line). Experimental conditions: λ_exc_ = 638 nm, *P* = 0.11 mW, *t* = 10 s.

The TERS spectra highlight a high contrast with respect to the signal acquired when the tip is removed from the near-field region of the sample, just excluding the feedback loop of the STM system. Vibrational bands of all molecules agree with the literature [60–63]. We finally apply our tips to obtain TERS spectra from the N-terminal domain of the *Escherichia coli* protein HypF (HypF-N). This a small stably folded α/β protein with 91-residues (10 kDa) [64] that is capable of forming amyloid species like those associated with neurological diseases such as Alzheimer’s and Parkinson’s [65], and have recently been the subject of TERS investigations [66–68]. In particular, we focus on the detection of toxic HypF-N oligomers that precede the formation of mature amyloid fibrils [69–70]. HypF-N oligomers (48 μM) are obtained by controlled aggregation (4 h, 25 °C, pH 5.5) of the HypF-N monomer in 50 mM acetate buffer, 12% (v/v) trifluoroethanol and 2 mM dithiothreitol [66]. The gold films are then immersed overnight in the oligomer solution, followed by rinsing in water to remove the excess of protein and then drying in air. TERS spectra are acquired with a tip featuring a tip radius *r*_tip_ = 15 nm. Figure 8 (red line) shows evidence of a strong TERS effect on oligomers, with the appearance of some of typical vibrational bands of protein samples such as the phenylalanine (Phe) ring breathing mode at 1004 cm^−1^ or the amide II band at 1550 cm^−1^ due to the C–N stretching mode and N–H bending mode of the atoms forming the peptide chain.

No signal is detected when the tip is not in contact with the surface Figure 8 (black line). After each TERS measurement, the tip is retracted from the sample and its emission is mapped in order to be sure the TERS signal does not come from molecules adsorbed on the tip apex.

### Evaluation of the enhancement factor

An estimation of the enhancement factor (EF) can be given by comparing the TERS signal increase with respect to the Raman signal measured when the tip is out of contact (far-field excitation conditions), normalizing to the different areas probed in each case [71]:

[5]
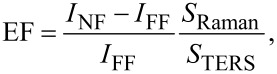


where *I*_NF_ is the near-field TERS signal, *I*_FF_ is the far-field Raman signal, *S*_TERS_ is the area probed when the tip is in contact and *S*_Raman_ is the area probed in far-field excitation conditions. *S*_TERS_ is calculated as the surface of the circle *S*_TERS_ = πr^2^_tip_ underneath the tip radius *r*_tip_. For the Raman signal, the probed area is calculated as the area of the elliptical intersection between the point spread function (PSF) of the objective (inclined by θ_inc_* =* 60° with respect to the vertical) and the horizontal plane. Considering *a* = *b* = λ/2·NA and *c* = 2λ/NA^2^ the semi-axes of the PSF, we find *S*_Raman_ = π*·a*·*c*’, where *a*’ *= a* ≈ 450 nm and 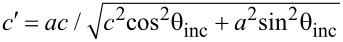
≈ 870 nm. Equation 5 provides meaningful results if *I*_FF_ ≠ 0. When no signal is detected in far-field conditions, we can still use the signal noise level (RMS) as a reference to estimate a lower bound of the EF. Measurements on HypF-N oligomers carried out with a tip whose radius of curvature has been observed by SEM to be *r*_tip_ = 15 nm show that indeed the EF is larger than 10^5^. Assuming the same tip radius for the other molecules we find EF ≥ 8 × 10^4^ for R6G, EF = 1.6 × 10^4^ for CV, EF ≥ 2.4 × 10^4^ for AZs, and EF ≥ 1.2 × 10^5^ for MB. These values compare well with the best results found in the literature [25,44].

### Assessment of the spatial resolution in TERS imaging

**Figure 9:**
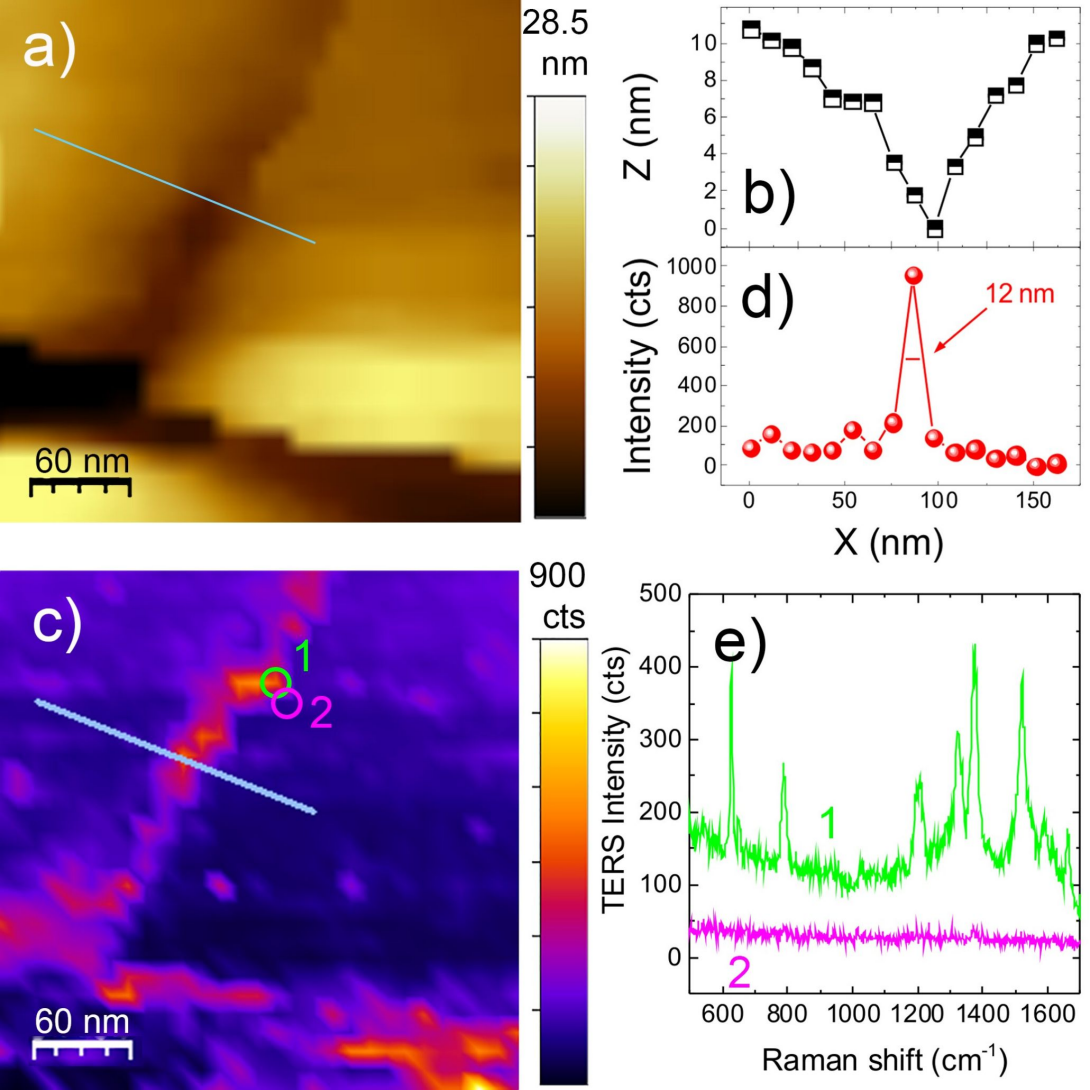
(a) STM image of Au(111) terraces on which R6G 10^−4^ M is adsorbed (∆*V* = 0.05 V – tip positive, current set point is 80 pA). The gray line indicates the zone where the line profile plotted in (b) is drawn. (c) Simultaneous TERS image at 1524 cm^−1^ (*P* = 1.0 mW, *t* = 0.5 s). The gray line indicates the zone where the line profile plotted in (d) is drawn. (e) TERS spectra acquired in correspondence with the circled areas in (c) taken at a distance of only one scanning step away from one another (10 nm).

Nanoscale resolution is shown in simultaneous morphological (STM) and chemical (TERS) mapping of R6G molecules (10^−4^ M) absorbed on Au(111). Experiments are carried out in gap-mode, with excitation at 638 nm. Figure 9a shows the STM topography acquired on a 300 × 300 nm^2^ area with step size of 10 nm. It displays adjacent gold terraces separated by trenches.

**Figure 10:**
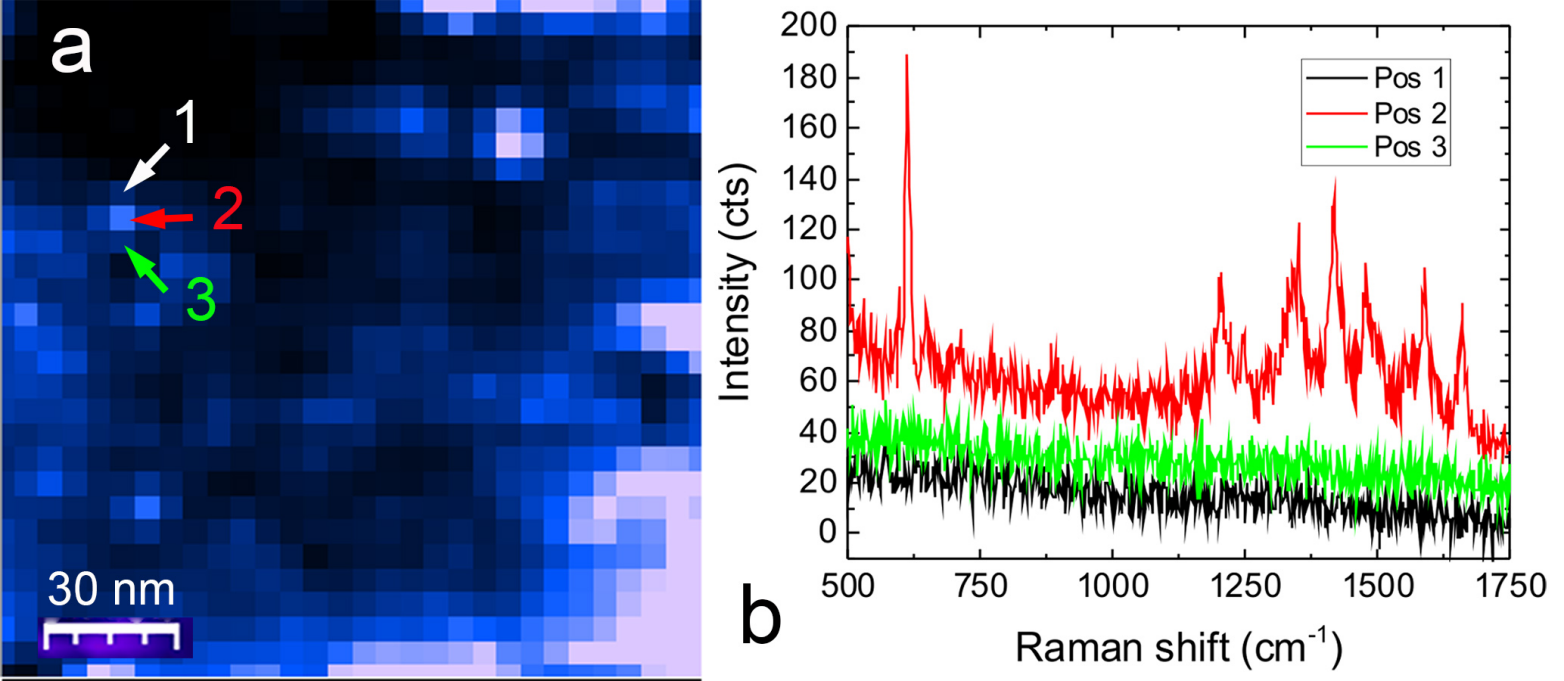
(a) TERS map and point spectra (b) acquired at the locations indicated with the labels 1, 2, 3, at a distance 5 nm from each other. Laser power 1 mW, integration time 0.5 s. The TERS signal in (a) is the integrated emission in the 1100–1500 cm^−1^ range.

The top diagonal trench has a depth of ≈10 nm and width of ≈50 nm at its largest point (line profile in Figure 9b). The bottom trench is approximately two times deeper. The TERS map in Figure 9c displays a strongly enhanced signal from the two trenches. This could in principle be due to a higher concentration of molecules in the channels, in combination with the presence of enhanced electric fields [72], caused by molecular diffusion into SERS-active sites [62]. Whatever the origin of the signal, we are able to map this effect with a spatial resolution equivalent to the scan step (10 nm). This is evident from the line profile of Figure 9d (drawn along the gray line in c), showing a full width at half maximum of 12 nm. The two spectra in Figure 9e, acquired at just one pixel distance from each other, corresponding to the circled areas in Figure 9c, highlight the possibility to probe the presence of R6G (green) or its absence (magenta) with 10 nm resolution. Furthermore, such a strong signal variability between two adjacent pixels, just 10 nm apart, suggests potential sub-pixel spatial resolution [72]. This conclusion is supported by TERS mapping of a 150 × 150 nm^2^ area (30 × 30 points) with step size of 5 nm (Figure 10a) in a different zone of the sample in which R6G absorbs in a patchy-like fashion. Spectra acquired on adjacent points (Figure 10b) show the capability to map the confinement of R6G molecules in a region smaller or equal to 5 nm consisting indeed of just a few molecules.

## Conclusion

In summary, we demonstrate a fast and inexpensive protocol to produce TERS tips by electrochemical etching of 125 µm diameter gold wires. The tips are robust and easy to manipulate. Their cost (1.05 €/tip) is 2.5 times cheaper than using standard 250 µm diameter wires, whereas the etching time (less than 2 min) is more than halved. 80% of the tips are TERS active. 50% of the tips have radius of curvature smaller than 35 nm. The tips have been tested on dyes, pigments and biomolecules showing enhancement factors ≈10^5^ (lower bound) in gap-mode. TERS maps of sub-monolayer films of R6G on Au(111) are shown with optical resolution better than 5 nm. The procedure can in principle be applied to thinner wires to further reduce costs and production times, although issues related to the fragility of the tip and difficulties in the manipulation and mounting have to be solved when working with very thin wires (e.g., 50 µm). Our protocol can be extended to other materials and requires minimal lab equipment and technical skills.

## Supporting Information

File 1 Picture of the TERS experimental setup.

File 2 A movie illustrating the electrochemical etching process.

## References

[1] Stöckle R M, Suh Y D, Deckert V, Zenobi R (2000). Chem Phys Lett.

[2] Anderson M S (2000). Appl Phys Lett.

[3] Hayazawa N, Inouye Y, Sekkat Z, Kawata S (2000). Opt Commun.

[4] Pettinger B, Picardi G, Schuster R, Ertl G (2000). Electrochemistry.

[5] Novotny L, Hecht B (2012). Principles of Nano-optics.

[6] Novotny L, van Hulst N (2011). Nat Photonics.

[7] Shi X, Coca-López N, Janik J, Hartschuh A (2017). Chem Rev.

[8] Yang Z, Aizpurua J, Xu H (2009). J Raman Spectrosc.

[9] Kottmann J P, Martin O J F, Smith D R, Schultz S J (2001). J Microsc (Oxford, U K).

[10] Barbry M, Koval P, Marchesin F, Esteban R, Borisov A G, Aizpurua J, Sánchez-Portal D (2015). Nano Lett.

[11] Zhang W, Yeo B S, Schmid T, Zenobi R (2007). J Phys Chem C.

[12] Sonntag M D, Klingsporn J M, Garibay L K, Roberts J M, Dieringer J A, Seideman T, Scheidt K A, Jensen L, Schatz G C, Van Duyne R P (2012). J Phys Chem C.

[13] Kharintsev S S, Hoffmann G G, Dorozhkin P S, With G d, Loos J (2007). Nanotechnology.

[14] Pettinger B, Ren B, Picardi G, Schuster R, Ertl G (2004). Phys Rev Lett.

[15] Hartschuh A, Sánchez E J, Xie X S, Novotny L (2003). Phys Rev Lett.

[16] Yano T-a, Verma P, Saito Y, Ichimura T, Kawata S (2009). Nat Photonics.

[17] Steidtner J, Pettinger B (2008). Phys Rev Lett.

[18] Zhang R, Zhang Y, Dong Z C, Jiang S, Zhang C, Chen L G, Zhang L, Liao Y, Aizpurua J, Luo Y (2013). Nature.

[19] Jiang S, Zhang Y, Zhang R, Hu C, Liao M, Luo Y, Yang J, Dong Z, Hou J G (2015). Nat Nanotechnol.

[20] Mauser N, Hartschuh A (2014). Chem Soc Rev.

[21] Wang X, Huang S-C, Huang T-X, Su H-S, Zhong J-H, Zeng Z-C, Li M-H, Ren B (2017). Chem Soc Rev.

[22] Sharma G, Deckert-Gaudig T, Deckert V (2015). Adv Drug Delivery Rev.

[23] Zhang Z, Sheng S, Wang R, Sun M (2016). Anal Chem.

[24] Zrimsek A B, Chiang N, Mattei M, Zaleski S, McAnally M O, Chapman C T, Henry A-I, Schatz G C, Van Duyne R P (2017). Chem Rev.

[25] Bonhommeau S, Lecomte S (2018). ChemPhysChem.

[26] Gucciardi P G, Trusso S, Vasi C, Patanè S, Allegrini M (2003). Appl Opt.

[27] Hell S W (2007). Science.

[28] Montgomery P C, Leong-Hoi A, Anstotz F, Mitev D, Pramatarova L, Haeberlé O (2016). J Phys: Conf Ser.

[29] Maouli I, Taguchi A, Saito Y, Kawata S, Verma P (2015). Appl Phys Express.

[30] Huang T-X, Huang S-C, Li M-H, Zeng Z-C, Wang X, Ren B (2015). Anal Bioanal Chem.

[31] Ren B, Picardi G, Pettinger B (2004). Rev Sci Instrum.

[32] Sasaki S S, Perdue S M, Perez A R, Tallarida N, Majors J H, Apkarian V A, Lee J (2013). Rev Sci Instrum.

[33] Bonaccorso F, Calogero G, Di Marco G, Maragò O M, Gucciardi P G, Giorgianni U, Channon K, Sabatino G (2007). Rev Sci Instrum.

[34] Taguchi A, Hayazawa N, Furusawa K, Ishitobi H, Kawata S (2009). J Raman Spectrosc.

[35] Kalbacova J, Rodriguez R D, Desale V, Schneider M, Amin I, Jordan R, Zahn D R T (2015). Nanospectroscopy.

[36] Yeo B-S, Zhang W, Vannier C, Zenobi R (2006). Appl Spectrosc.

[37] Asghari-Khiavi M, Wood B R, Hojati-Talemi P, Downes A, McNaughton D, Mechler A (2012). J Raman Spectrosc.

[38] Rodriguez R D, Sheremet E, Müller S, Gordan O D, Villabona A, Schulze S, Hietschold M, Zahn D R T (2012). Rev Sci Instrum.

[39] Saito Y, Murakami T, Inouye Y, Kawata S (2005). Chem Lett.

[40] Brejna P R, Griffiths P R (2010). Appl Spectrosc.

[41] De Angelis F, Das G, Candeloro P, Patrini M, Galli M, Bek A, Lazzarino M, Maksymov I, Liberale C, Andreani L C (2010). Nat Nanotechnol.

[42] Farahani J N, Pohl D W, Eisler H-J, Hecht B (2005). Phys Rev Lett.

[43] Fleischer M, Weber-Bargioni A, Altoe M V P, Schwartzberg A M, Schuck P J, Cabrini S, Kern D P (2011). ACS Nano.

[44] Fujita Y, Walke P, De Feyter S, Uji-i H (2016). Jpn J Appl Phys.

[45] Eisele M, Krüger M, Schenk M, Ziegler A, Hommelhoff P (2011). Rev Sci Instrum.

[46] Lopes M, Toury T, de La Chapelle M L, Bonaccorso F, Giuseppe Gucciardi P (2013). Rev Sci Instrum.

[47] James D F (1974). J Fluid Mech.

[48] Baykul M C (2000). Mater Sci Eng, B.

[49] Wang X, Liu Z, Zhuang M-D, Zhang H-M, Wang X, Xie Z-X, Wu D-Y, Ren B, Tian Z-Q (2007). Appl Phys Lett.

[50] Foti A, D’Andrea C, Bonaccorso F, Lanza M, Calogero G, Messina E, Maragò O M, Fazio B, Gucciardi P G (2013). Plasmonics.

[51] Qian H, Hsu S-W, Gurunatha K, Riley C T, Zhao J, Lu D, Tao A R, Liu Z (2018). Nat Photonics.

[52] Mertens J, Kleemann M-E, Chikkaraddy R, Narang P, Baumberg J J (2017). Nano Lett.

[53] Pettinger B, Domke K F, Zhang D, Schuster R, Ertl G (2007). Phys Rev B.

[54] Wang X, Braun K, Zhang D, Peisert H, Adler H, Chassé T, Meixner A J (2015). ACS Nano.

[55] Carles R, Bayle M, Benzo P, Benassayag G, Bonafos C, Cacciato G, Privitera V (2015). Phys Rev B.

[56] Hugall J T, Baumberg J J (2015). Nano Lett.

[57] Sanders A, Bowman R W, Zhang L, Turek V, Sigle D O, Lombardi A, Weller L, Baumberg J J (2016). Appl Phys Lett.

[58] Schweppe H, Winter J, West Fitzhugh E (1997). Madder and Alizarin. Artists’ Pigments.

[59] Chiti F, Dobson C M (2006). Annu Rev Biochem.

[60] Cañamares M V, Chenal C, Birke R L, Lombardi J R (2008). J Phys Chem C.

[61] Kudelski A (2005). Chem Phys Lett.

[62] Holmgren A, Wu L, Forsling W (1999). Spectrochim Acta, Part A.

[63] D’Andrea C, Fazio B, Gucciardi P G, Giordano M C, Martella C, Chiappe D, Toma A, Buatier de Mongeot F, Tantussi F, Vasanthakumar P (2014). J Phys Chem C.

[64] Tatini F, Pugliese A M, Traini C, Niccoli S, Maraula G, Ed Dami T, Mannini B, Scartabelli T, Pedata F, Casamenti F (2013). Neurobiol Aging.

[65] Campioni S, Mannini B, Zampagni M, Pensalfini A, Parrini C, Evangelisti E, Relini A, Stefani M, Dobson C M, Cecchi C (2010). Nat Chem Biol.

[66] van den Akker C C, Deckert-Gaudig T, Schleeger M, Velikov K P, Deckert V, Bonn M, Koenderink G H (2015). Small.

[67] Deckert-Gaudig T, Kurouski D, Hedegaard M A B, Singh P, Lednev I K, Deckert V (2016). Sci Rep.

[68] Kurouski D, Deckert-Gaudig T, Deckert V, Lednev I K (2014). Biophys J.

[69] Cleary J P, Walsh D M, Hofmeister J J, Shankar G M, Kuskowski M A, Selkoe D J, Ashe K H (2005). Nat Neurosci.

[70] D'Andrea C, Foti A, Cottat M, Banchelli M, Capitini C, Barreca F, Canale C, de Angelis M, Relini A, Maragò O M (2018). Small.

[71] Patane S, Gucciardi P G, Labardi M, Allegrini M (2004). Riv Nuovo Cimento Soc Ital Fis.

[72] Bhattarai A, Joly A G, Hess W P, El-Khoury P Z (2017). Nano Lett.

