# Supplementary figures and images for: Low cost tips for tip-enhanced Raman spectroscopy fabricated by two-step electrochemical etching of 125 µm diameter gold wires
Source: Beilstein J Nanotechnol. 2018 Oct 22;9:2718–29. doi: 10.3762/bjnano.9.254 (PMC6204785; doi:10.3762/bjnano.9.254)

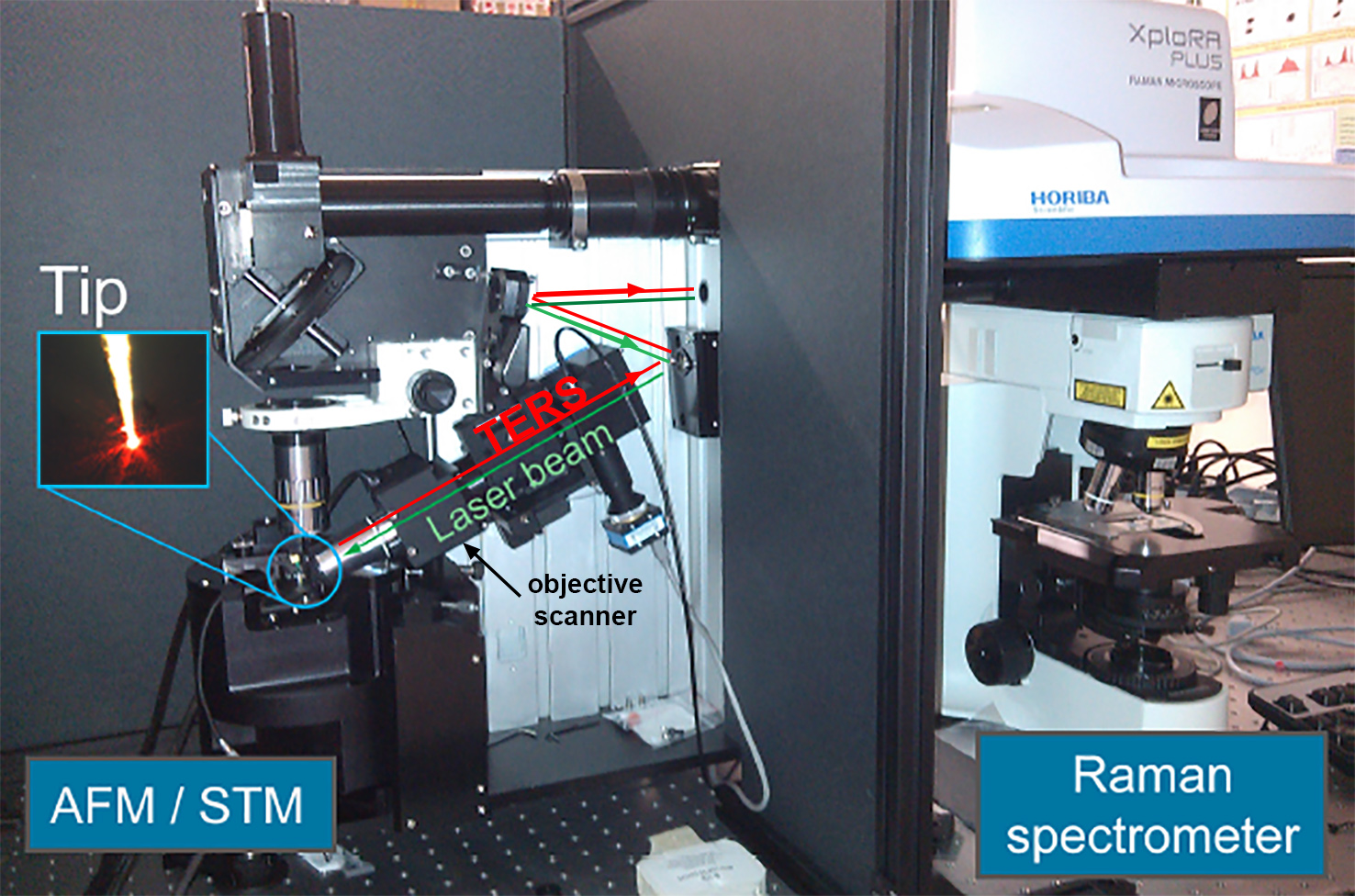

Supplement: File 1 — Picture of the TERS experimental setup. [file Beilstein_J_Nanotechnol-09-2718-s001.png]
